# Report of Case Involving Loss of Corresponding Portions of Both Hemispheres of the Brain

**Published:** 1857-12

**Authors:** L. N. Dimmick

**Affiliations:** Freedom, Lasalle Co., Illinois


### ARTICLE IV.

### REPORT OF CASE INVOLVING LOSS OF CORRESPONDING PORTIONS OF BOTH HEMISPHERES OF THE BRAIN.

BY L. N. DIMMICK, M.D., FREEDOM, LASALLE CO., ILLINOIS.

While there exists such a difference in theories with regard to the physiological action of the different portions of the encephalon, it is desirable that all facts having a direct bearing upon this subject should be candidly observed and recorded.

On the 22d August, 1852, I was called to the house of Mr. George Barnard, town of Adams, Lasalle Co., Ill., to see his child, a boy, two years and six months old, who had been kicked by a colt on the head, and found him in violent convulsions. The kick was received about the middle of the frontal portion of the frontal bone, fracturing it laterally from one temporal fossa to the other. At the lower edge of the fracture the frontal bone was depressed upon the brain not less than half an inch. The meningeal membranes were severed, and portions of the skull driven into the substance of the brain.

From a late conversation with his mother, I learn that he was hurt on the 18th, just four days previous to my first visit. During that time had appeared so well, that it was supposed that the skull was not fractured, that the wound extended only through the scalp. The first evening he vomited a little, but the three succeeding days he played around the house as usual, rode a stick calling it his horse, appeared a little more restless than before the injury; the evening of the 21st complained of being sick, the pupils of his eyes were dilated. On the 22d, was taken with convulsions, which continued with great violence for six hours. After elevation of the depressed bone, removal of both tables of the skull from the substance of the brain, and free discharge of purulent matter, mixed with blood and portions of the brain, the boy became rational. This was on the 23d.

His right side was found to be paralyzed on the subsidence of his convulsions, and remained so as long as he survived. For two or three days the suppuration and discharge of sloughed portions of the brain was profuse, accompanied at times by copious hemorrhage, so that by about the 25th or 26th, the anterior portion of the frontal lobes of both hemispheres, as far back I judged as the corpus callosum, was entirely gone. The orbital plates were laid bare. After washing out the cavity the brain could be seen and felt, presenting an irregular surface or wall, nearly perpendicular to the base of the skull. And yet, to my surprise, he retained his mental faculties apparently unimpaired. During the greater part of the remaining time he was perfectly rational. When not spoken to, or his mind apparently not occupied by things around him, he frequently kept up a monotonous hum or song.

The portion sloughed involved, according to the phrenological system, the perceptive faculties—causality, comparison, etc.— I had the curiosity to question him closely from day to day to test his capacity in this respect, and yet in none could I discover but that he manifested his former perception and reasoning powers.

A brief sketch of this case, written in answer to a letter of inquiry, was unexpectedly to me, published in the *American Phrenological Journal* for 1857, in the June number. Fowler, in attempting to reconcile this and similar cases with his phrenological theories, shrewdly attempts to soften down this "rather strong language." He says this ability (manifesting his former reasoning powers) was destroyed by his loss of brain. That "whatever is presented to his senses awakens old impressions," but excites no new ideas, leads to no new mental results, increases not in the least the stock of knowledge or ideas entertained up to the time of the accident.

In questioning him on points requiring the exercise of comparison, causality, eventuality, numeration, form, color, etc., I asked him questions that I do not think could ever have occurred to him, and endeavored to confuse and puzzle him, but his apt replies and his judgment satisfied me that he was more precocious than most children of his age.

That the reader may judge for himself, I will state some circumstances noted down during their relation by his mother. He was very particular about his watchers; on the evening of the 29th he was told that one of them was his grandmother; he turned his eyes towards her, and then replied, with evident disappointment, "No! that ain't *my* grandmother."

He had been fond of seeing himself in the mirror; while his wound was uncovered it was carried to him; he looked in it, and screaming with fright, pushed it from him.

He would take a bush and keep the flies from him. Once, when his mother took it to drive them away, he remarked, "Mother, you won't whip me, will you? Whip Peter (his brother), he is naughty," and then specified the wrong act.

On the 30th he lost the power of speech, still remaining, in other respects, nearly the same. He had a desire for food, and by signs called for it and other things that he wanted. On the 4th of September his grandmother came to see him; he expressed by his kindling eyes, his smiles and his attempts to talk, his great pleasure at seeing her.

On the 7th of September he was evidently sinking rapidly. He gradually became comatose, and died on the 8th, just three weeks after receiving the injury. The natural action of the bowels and bladder continued throughout the whole time.

